# Does Vitamin D Deficiency Affect the Immunogenic Responses to Influenza Vaccination? A Systematic Review and Meta-Analysis

**DOI:** 10.3390/nu10040409

**Published:** 2018-03-26

**Authors:** Ming-Dar Lee, Chao-Hsu Lin, Wei-Te Lei, Hung-Yang Chang, Hung-Chang Lee, Chun-Yan Yeung, Nan-Chang Chiu, Hsin Chi, Jui-Ming Liu, Ren-Jun Hsu, Yu-Jyun Cheng, Tzu-Lin Yeh, Chien-Yu Lin

**Affiliations:** 1 Department of Pediatrics, Hsinchu MacKay Memorial Hospital, Hsinchu 300, Taiwan; 4554@mmh.org.tw (M.-D.L.); 3099@mmh.org.tw (C.-H.L.); lazyleisure@gmail.com (W.-T.L.); 4569@mmh.org.tw (Y.-J.C.); 5767@mmh.org.tw (T.-L.Y.); 2 Department of Pediatrics, MacKay Children’s Hospital, Taipei 104, Taiwan; drchy@seed.net.tw (H.-Y.C.); ped2435@mmh.org.tw (H.-C.L.); cy.yeung@mmh.org.tw (C.-Y.Y.); ncc88@mmh.org.tw (N.-C.C.); chi.4531@mmh.org.tw (H.C.); 3 Graduate Institute of Life Sciences, National Defense Medical Center, Taipei 114, Taiwan; mento1218@gmail.com (J.-M.L.); hsurnai@gmail.com (R.-J.H.); 4 Division of Urology, Department of Surgery, Taoyuan General Hospital, Ministry of Health and Welfare, Taoyuan 330, Taiwan; 5 Department of Medicine, National Yang-Ming University, Taipei 112, Taiwan

**Keywords:** influenza, influenza vaccination, vitamin D, 25(OH)D, calcitriol, human health

## Abstract

Influenza virus infection is a major global public health problem, and the efficacy of influenza vaccination is not satisfactory. Vitamin D is involved in many immune-mediated inflammatory processes. The impact of vitamin D levels on the immunogenic response to influenza vaccination is not clear. We performed a comprehensive literature search and systematic review of studies that investigated vitamin D and influenza vaccination. Data pertaining to study population, vaccine components, vitamin D levels, and immunogenic response were analyzed. Nine studies, with a combined study population of 2367 patients, were included in the systematic review. Four studies were included in the meta-analysis to investigate the influence of vitamin D deficiency (VDD) on the seroprotection (SP) rates and seroconversion (SC) rates following influenza vaccination. We found no significant association between vitamin D level and the immunogenic response to influenza vaccination. However, strain-specific differences may exist. We observed lower SP rates of influenza A virus subtype H3N2 (A/H3N2) and B strain in VDD patients than patients with normal vitamin D levels (A/H3N2: 71.8% vs. 80.1%, odds ratio (OR): 0.63, 95% confidence interval (CI): 0.43–0.91, *p* = 0.01; B strain: 69.6% vs. 76.4%, OR: 0.68, 95% CI: 0.5–0.93, *p* = 0.01). However, the SP rates of A/H1N1 and SC rates of all three strains were not significantly different in VDD and control groups. In conclusion, no association was observed between VDD and immunogenic response to influenza vaccination.

## 1. Introduction

Influenza is an important infectious disease and a major contributor to the global disease burden. It is estimated to be responsible for 250,000–500,000 deaths annually, especially among the elderly [1]. Influenza vaccination is widely used to prevent influenza infection; however, the vaccine efficacy is unsatisfactory. The pooled efficacy in adults aged 18–65 years was 59% in a meta-analysis published in 2012; however, there was no evidence of protection observed in the elderly group [2]. Protection by influenza vaccine is inadequate, and breakthrough infection is common, which results in influenza-related morbidity and mortality. Refinement has been made to enhance the immune response to influenza vaccines, such as by providing additional adjuvant supplements, nutritional interventions or by increasing the vaccine dose [3,4]. The composition of the influenza vaccine usually contains split virions with two A strains (influenza A virus subtype H1N1 and H3N2: A/H1N1 and A/H3N2) and one B strain (Victoria or Yamagata lineages). The antigen immunogenic response to B strain was shown to be poorer in previous studies when comparing with A/H1N1 and A/H3N2 strains. [5,6]. To summarize, the protective efficacy of the currently used influenza vaccine is still under satisfactory.

Vitamin D plays an important role in bone health. The association of vitamin D deficiency (VDD) with osteoporosis and rickets is well documented [7]. In addition, vitamin D has also been shown to be involved in inflammatory processes in the context of several diseases [8]. Vitamin D receptors are present in various tissues, and several studies have found an association between vitamin D and systemic diseases [9,10,11,12,13]. For example, VDD was associated with higher risk of type 1, type 2 diabetes and metabolic syndromes [14]. Vitamin D receptors were found in cardiomyocytes and arterial wall cells and increased risks of cardiovascular disease and ischemic stroke were found in patients with VDD [15]. A higher risk of asthma was found in patients with VDD [11]. Compared with healthy controls, lower vitamin D levels were noted in patients with systemic sclerosis [10]. Furthermore, patients with VDD were shown to have a higher risk of all-cause mortality, and more than half of all children admitted to intensive care units were shown to have VDD [9,12,16]. Association between VDD and cancers has also been explored; patients with VDD were shown to have a higher risk of colorectal cancer [17,18,19]. A higher risk of influenza infection and childhood asthma has been reported in patients with VDD [11,20]. Supplementation of vitamin D may prevent asthma exacerbation and improve winter-related atopic dermatitis [21,22]. However, the definite mechanism underlying this association and the role of vitamin D remains largely unclear [23,24].

VDD is common worldwide; however, its prevalence varies according to geographical location, latitude, race and seasons [25,26]. Both influenza infection and VDD are more common in cold seasons [25,26,27]. Individuals with VDD were shown to have a higher risk of influenza infection [20]. Vitamin D is known to possess immunomodulatory effects and might affect the immunogenic response to influenza vaccination. VDD may contribute to the low efficacy of influenza vaccination. However, the results of studies that have investigated vitamin D and influenza vaccination have been largely discrepant and inconclusive. Therefore, this systematic review and meta-analysis was conducted to evaluate the impact of VDD on immune response to influenza vaccination.

## 2. Materials and Methods

### 2.1. Study Design and Study Selection

This study was approved by the Ethics Committee of the MacKay Memorial Hospital, Taiwan (registry No.: 16MMHIS174e). Our systematic review and meta-analysis was conforming with the Preferred Reporting Items for Systematic Review and Meta-Analysis Protocols guidelines [28]. “Influenza vaccine,” “vitamin D,” “25-hydroxyvitamin D,” and “calcitriol” were the key terms used for literature search. Keywords were combined when using Boolean searches, and the search was performed using keywords, Boolean operators, and MeSH descriptors. The detailed search strategy is shown in Table S1. A systematic literature search was performed in the following online databases: Embase; PubMed; the Cochrane Library; the Cumulative Index to Nursing and Allied Health (CINAHL); the Airiti Library (Art Image Indexing Service on the Internet Database); and the National Digital Library of Theses and Dissertations in Taiwan (NDLTD). All studies published as of December 2017 were eligible for inclusion. The Cochrane Collaboration Central Register of Controlled Clinical Trials, Cochrane Systematic Reviews, and ClinicalTrials.gov databases were searched manually for additional references. Two authors (M.-D.L. and C.-Y.L.) conducted the search independently, while disagreements were resolved through discussion with the third author (C.-H.L.).

Two independent reviewers (M.-D.L. and C.-Y.L.) assessed the eligibility of each paper after the initial search was conducted. The inclusion criteria were (1) inclusion of a controlled group in the study design; (2) administration of influenza vaccination; (3) measurement or supplementation of vitamin D; (4) reporting of at least one immunological response to influenza vaccination. The following criteria were excluded: (1) articles irrelevant to the topic; (2) duplicate publications; (3) trials with a crossover study design; (4) animal studies; and (5) case reports and studies which excluded a controlled group.

### 2.2. Data Extraction and Quality Assessment

Two authors (M.-D.L. and C.-Y.L.) used the Cochrane Review risk of bias assessment tool independently to evaluate the quality of all eligible articles. Each publication was assessed with its adequacy of randomization, allocation concealment, blinding methods, implementation of the intent-to-treat analysis, dropout rate, complete outcomes data, selective data reporting, and other potential biases.

The articles were scrutinized, and the data pertaining to the following variables were extracted: study population; influenza vaccine components; vitamin D levels; supplementation of vitamin D; details of vaccine immune responses; and adverse effects. Discrepancies between the two independent evaluations for potential articles were determined through discussion and consensus. The primary outcome was seroprotection (SP) of influenza vaccination, which was defined as antibody titer after vaccination ≥40 as measured by micro-neutralization. The secondary outcome was seroconversion (SC), which was defined as a fourfold increase in antibody titer after influenza vaccination. The SP and SC were assessed based on the hemagglutination inhibition (HI) antibody titers. The HI antibody titer that equals the maximum dilution capable of inhibiting the agglutination of guinea pig red blood cells was with the influenza viruses under standardized conditions [29]. Other comparative variables contained the components of the vaccine, the prevalence of VDD, vitamin D supplementation, and severe adverse effects.

### 2.3. Data Synthesis and Analysis

In order to determine differences in the efficacy of influenza vaccination in VDD groups and control groups, the authors extracted, analyzed and compared the immunogenicity data from all the studies. A random effects model was employed due to the significant (and expected) heterogeneity among the studies [30]. The outcomes are presented as point estimates with 95% confidence intervals (CIs). *I*-square and Cochran’s *Q* tests were used to test the heterogeneity across studies. A *p*-value < 0.10 for chi-squared test of the *Q* statistic or an *I*-square > 50% was considered indicative of statistically significant heterogeneity [31]. To observe the effect on the overall results, a sensitivity analysis was performed by repeating the analysis after sequential exclusion of one study at a time. Potential publication bias was assessed through the observation of the symmetry of funnel plots and the use of Egger’s test [32]. Review Manager (version 5.3.5, Cochrane Community: Lisbon, Portugal, available online: http://community.cochrane.org/tools/review-production-tools/revman-5/revman-5-download) was used for our analyses.

## 3. Results

### 3.1. Description of Studies and Quality Assessment

**Figure 1:**
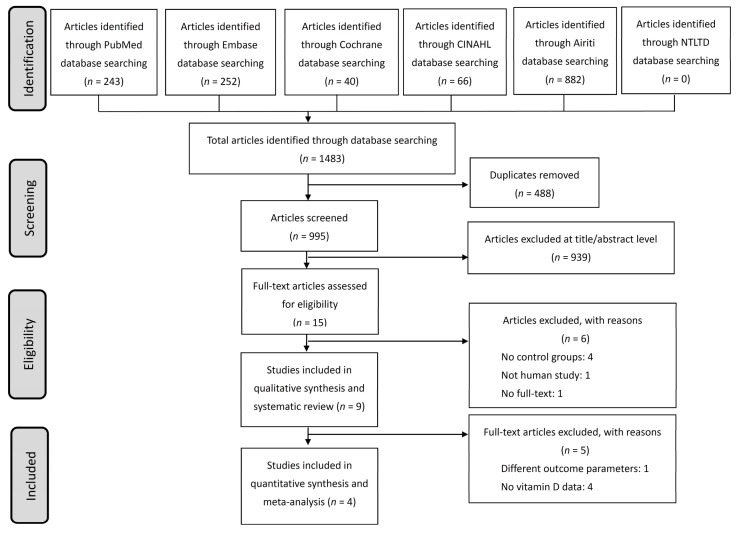
Schematic illustration of the literature search and the study-selection criteria. CINAHL = the Cumulative Index to Nursing and Allied Health; Airiti = Art Image Indexing Service on the Internet Database; NTLTD = the National Digital Library of Theses and Dissertations in Taiwan.

The flowchart schematic illustration of the literature search and study-selection criteria is presented in Figure 1. Nine publications were included in our qualitative synthesis and critical review after the examination. (Table 1) [33,34,35,36,37,38,39,40,41]. Two trials conducted during different influenza seasons with different patient numbers, vaccine components, and years of study were published in the same article and regarded as two independent studies [37]. Consequently, nine publications compromising ten studies were enrolled in our systematic review. Most studies (7/10) were conducted in the USA, among which three studies were performed in children. Two studies involved (human immunodeficiency virus) HIV-infected individuals, and one study investigated influenza vaccination in patients with prostate cancer [35,39,40]. Trivalent inactivated influenza vaccine (TIV) was administered in most studies (9/10). In total, 2367 patients were enrolled in these studies; the female-to-male ratio was 1:1.14. Vitamin D levels were measured in eight studies, and the percentage of patients with VDD varied widely between different studies (0.9–45%). The cutoff values used to determine VDD were 20 ng/mL in three studies and 25 ng/mL in the other three studies. Vitamin D supplementation was performed in four studies [38,39,41]. 

The Cochrane assessment tool shows that most of the included studies had a low potential for bias. The detailed quality assessment of each included study is presented in Table S2.

### 3.2. Data Synthesis and Meta-Analysis

Based on the basis of currently available evidence, no association was found between VDD and the immunogenic response to influenza vaccination (Table 1). Positive association was found in two studies, and negative association was found in two studies. VDD was not associated with the immunogenic response in the other five studies. Data pertaining to immunogenic response including SP and SC following influenza vaccination were extracted for further meta-analysis. Ultimately, four studies with a combined study population of 1517 patients were included in our meta-analysis [35,36,37,38]. Data pertaining to vitamin D levels were unavailable for these studies, and therefore these studies were not eligible for the meta-analysis [34,39,40,41]. High baseline SP rate (75%) of enrolled individuals was reported in one study [34]. One study was excluded in the meta-analysis because of different outcome parameters [33]. In Crum-Cianflone’s study, the study population was classified into two groups (HIV-infected and HIV-uninfected), and the groups were compared separately [35]. Sundaram’s study was conducted in two different seasons with different influenza vaccines and was regarded as different studies [37]. Finally, four publications with six studies were enrolled in the final meta-analysis.

**Figure 2:**
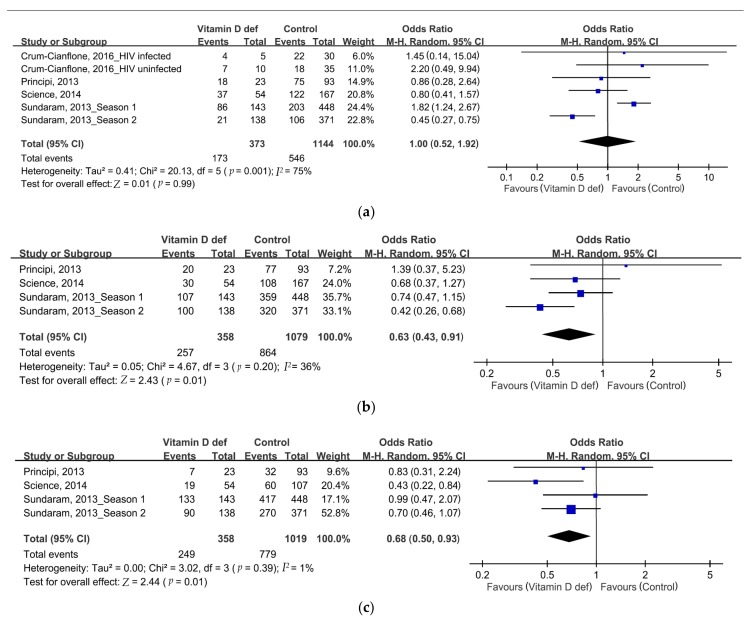
Forrest plot of the SP rates of influenza vaccination in the VDD (vitamin D deficiency) and placebo groups. (**a**) Strain A/H1N1; (**b**) strain A/H3N2; (**c**) strain B. df = degrees of freedom; def = deficiency; M-H = Mantel-Haenszel.

**Figure 3:**
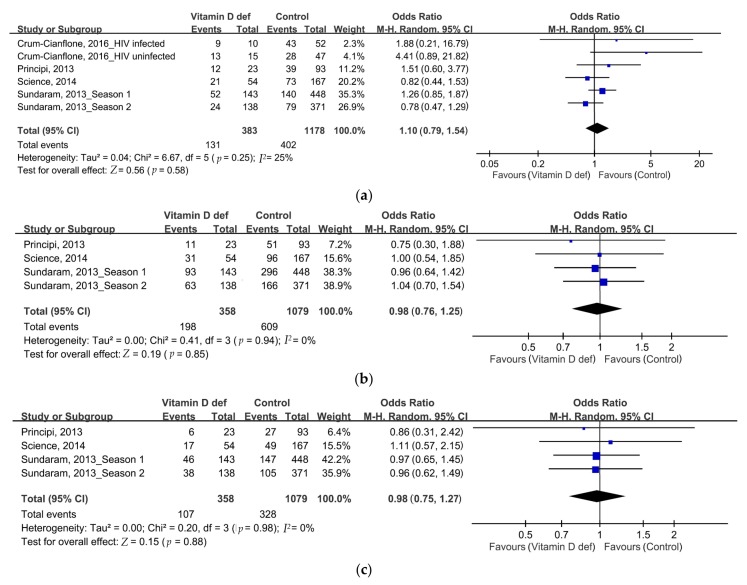
Forrest plot of the seroconversion (SC) rates of influenza vaccination in the VDD and placebo groups. (**a**) Strain A/H1N1; (**b**) strain A/H3N2; (**c**) strain B.

By comparing the SP rates after influenza vaccination, no differences of SP rates of strain A/H1N1 were noted (46.4% vs. 47.7%, odds ratio (OR): 1, 95% CI: 0.52–1.92, *p* = 0.99, *I*^2^ = 175%, Figure 2a). However, we found significantly lower SP rates of strain A/H3N2 and strain B in the VDD group than in patients with normal vitamin D levels (strain A/H3N2: 71.8% vs. 80.1%, OR: 0.63, 95% CI: 0.43–0.91, *p* = 0.01, *I*^2^ = 36%, Figure 2b; B strain: 69.6% vs. 76.4%, OR: 0.68, 95% CI: 0.5–0.93, *p* = 0.01, *I*^2^ = 1%, Figure 2c). No significant differences were observed between the three strains with respect to the SC rates after influenza vaccination (Figure 3a–c). The SP and SC rates are summarized in Table 2. No serious adverse effects were reported. The funnel plots were also assessed.

## 4. Discussion

Our systematic review found no association between VDD and the immunogenic response to influenza vaccination based on the currently available evidence. However, strain-specific differences may exist, and decreased SP rates of A/H3N2 and B strain were observed in patients with VDD (6.8–8.3% difference in SP rates with low heterogeneity). VDD may decrease the immunogenic response to influenza vaccination, and correction of VDD may potentially improve the protective efficacy of influenza vaccination. Further studies are warranted to elucidate the influence of VDD on individual vaccine strains and the impact of vitamin D supplementation.

Influenza is a highly contagious disease characterized by widespread antigenic variations. Although the influenza vaccination is widely used nowadays, it remains an important health threat worldwide. The effectiveness of current influenza vaccination is not adequate, and protection rates in elderly people are probably as low as 30% [2,42,43]. Multiple factors are responsible for the low protective efficacy; these include antigen drift, seasonal mismatch, and manufacture technique limitations [2,44,45]. Immunosenescence, gradual deterioration of the immune system brought on by natural aging, poor nutritional status, and higher rates of comorbid diseases also play important roles in the hyporesponsiveness of influenza vaccination, especially in the elderly [46]. Efforts have been made to improve the immune response to influenza vaccination. One commonly used strategy involves the use of adjuvants [47]. Additionally, TIV with high doses (four times the standard dose) and intradermal injection were shown to induce significantly higher antibody response; however, these strategies are not widely used [3]. Nutritional interventions (e.g., use of probiotics) may provide a simple, convenient and practical solution [48,49]. VDD may affect the immune response to influenza vaccination, and correction of VDD may help enhance the humoral response. Our results suggest that vitamin D levels do not affect the immunogenicity of influenza vaccination and routine vitamin D supplementation is not recommended. However, VDD may attenuate the immunogenic response to specific strains, such as strain A/H3N2 and strain B. Compared with strains A/H1N1 and A/H3N2, the immunogenic response to strain B was shown to be poorer in previous reports [5,6]. The immunogenic responses were inconsistent between different vaccine strains and variations of pre-vaccinated antibody levels may contribute to the observed differences. Sometimes, one of the vaccine strains may be included in the following years, therefore booster effects may interfere the immunogenic responses to the same strain. For example, A/California/7/2009 (H1N1) has been included in the suggested vaccine component every year between 2010–2017 but the accompanying strains A/H3N2 and B have changed every year. Differences in immunogenic response to vaccination with different component strains of influenza were found; however, the role of strain difference remains unclear. Our systematic review showed that strain A/H3N2 had the highest SP and SC rates (Table 2). Further studies are warranted to clarify the influence of vitamin D on the individual strain components.

In addition to its important role in bone metabolism, the anti-inflammatory properties of vitamin D have been investigated in the last decade [7,8]. Vitamin D receptors are present in many systems, and vitamin D was found to play important roles in various diseases. Vitamin D is not only a steroid hormone but also an important immune modulator. Patients with VDD were shown to have a higher risk of several cancers [17,19]. VDD is also prevalent in patients with critical illnesses and may contribute to death because of cardiovascular disease, cancer and other causes [9,12,16,50]. Supplementation of vitamin D may help prevent these diseases or improve treatment outcomes. Increased risk of infection with influenza virus in patients with VDD has been observed as well [20]. However, the underlying mechanisms are not well characterized. Both VDD and influenza infection are more common in cold seasons. Lower solar radiation may contribute to VDD and affect the susceptibility to influenza infection. Vitamin D may also alter the immune response to influenza infection. Additionally, VDD may affect the effectiveness of influenza vaccination and increase the risk of influenza infection. Our systematic review found no obvious association between VDD and immunogenicity of influenza vaccine. Concomitant supplementation of vitamin D during influenza vaccination is not needed from this viewpoint.

Although the importance of vitamin D has been recognized in recent years, there is no clear consensus on the definition of VDD [51,52,53]. Generally, vitamin D supplementation is recommended for pregnant women and newborns [54]. The cutoff values of vitamin D levels used to define VDD are not standardized, and different definitions of VDD are recommended by different societies. On the basis of the concentration of 25(OH)D, a serum concentration of 50 nmol/L (20 ng/mL) is sufficient for 97% of the population according to the recommendations of the Institute of Medicine published in 2010 [52,54]. The cutoff level recommended by the Endocrine Society is 30 ng/mL [53]. Similarly, a cutoff level of 20 ng/mL was adopted in half of the enrolled studies, and 25 ng/mL was used in the other studies (Table 1). Use of different definitions may result in different outcomes and interfere with the comparison of response rates. Furthermore, the prevalence of VDD tends to vary in different countries [25,26,27]. A similar phenomenon was also observed in our systematic review. The reported prevalence of VDD varied widely in the different studies (range, 0.9–45%). Moreover, vitamin D supplement was administered in three studies. The recommended daily dosage in children is 400–1000 IU [52,53,54]. However, single-day high-dose vitamin D therapy (stoss therapy) has also been investigated. Further studies are required to investigate the optimal dosage and protocol for vitamin D supplementation to enhance the immunogenic response to influenza vaccination. 

Our study had some limitations. First, much heterogeneity was observed with respect to the study design, study participants, and the study period. The cutoff values of VDD were not the same among the studies, and data pertaining to vitamin D levels were not available for all studies. Therefore, not all the enrolled studies were eligible for meta-analysis, and further large-scale studies are warranted to confirm our findings. Second, the prevalence of VDD varied widely among the studies, and the causation of VDD is multifactorial. Presence of risk factors for VDD may interfere with the immunogenic response to influenza vaccination as well. Furthermore, although SP rates and SC rates are regarded as reliable parameters for evaluation of immunogenicity of influenza vaccination, the pre-vaccination baseline antibody titers were not similar in the included studies. Vitamin D supplementation was not administered in all studies. Moreover, the components of the influenza vaccine and the prevalent influenza strains were different each year. Ideally, it is more valuable to compare the immunogenic response in a similar study population with different vitamin D levels, but with similar pre-vaccination baseline antibody titer, the same vitamin D supplementation, and the same influenza vaccine. 

## 5. Conclusions

Our study suggests that VDD is not associated with the immunogenic response to influenza vaccination based on the currently available data. Routine correction of VDD to enhance the immunogenicity of influenza vaccination is not recommended. However, strain-specific differences may exist, and VDD may attenuate the immune response to strain A/H3N2 and strain B following influenza vaccination. Adjuvant vitamin D supplementation may improve the immunogenic response to specific strains. Further studies are warranted to elucidate the influence of VDD on individual vaccine strains and the impact of vitamin D supplementation.

## Figures and Tables

**Table 1:** Characteristics of randomized controlled trials investigating vitamin D and influenza vaccination.

Studies Author, Year	Country	Participants (M%:F%)	Age (y/o) Mean (SD)	Vit D cutoff 25-OH D level (ng/ml)	VDD Patients (%)	Supplement of Vit D	Type of Vaccine	Components of Vaccine	Measured Outcomes	Associated with VDD
Lin, 2017 [33]	USA	135 children (46%:54%)	NR	20	61 (45%)	No	LAIV (83) TIV (52)	A/California/7/2009 (H1N1); A/Texas/50/2012 (H3N2); B/Massachusetts/2/2012	Antibody titers	Weakly negative
Sadarangani, 2016 [34]	USA	159 elders (61.6%:38.4%)	59.5 (median)	25	8 (5%)	No	TIV	A/California/7/2009 (H1N1); A/Perth/16/2009 (H3N2); B/Brisbane/60/2008	Antibody titers	Weakly positive
Crum-Cianflone, 2016 * [35]	USA	64 HIV-infected (93.8%:6.2%); 64 HIV-uninfected (89.1%:10.9%)	35; 34.5	20	16 (25%); 11 (17.2%)	No	Monovalent inactivated vaccine	A/California/7/2009(H1N1)	Seroconversion Antibody titers	No
Science, 2014 * [36]	Canada	221 children (48%:52%)	9.16	25	2 (0.9%)	No	TIV	A/Brisbane/59/2007 (H1N1); A/Brisbane/10/2007 (H3N2); B/Florida/4/2006.	Antibody titers Seroprotection	No
Sundaram, 2013, Season 1 * [37]	USA	591 adults (39%:61%)	64 ± 10 years	25	143 (29%)	No	TIV	A/Brisbane/59/2007 (H1N1); A/Brisbane/10/2007 (H3N2); B/Florida/4/2006	Seroprotection	Negative
Sundaram, 2013, Season 2 * [37]	USA	509 adults (38%:62%)	66 ± 10	25	138 (27%)	No	TIV	A/Brisbane/59/2007-like (H1N1), A/Brisbane/10/2007-like (H3N2), B/Brisbane/60/2008-like; A/California/7/2009 (H1N1)	Seroprotection	No
Principi, 2013 * [38]	Italy	116 AOM children (52.6%:47.4%)	3 ± 1	20	23 (19.8%)	Yes	TIV	A/California/7/2009(H1N1); A/Perth/16/2009(H3N2); B/Brisbane/60/2008	Seroprotection Seroconversion Antibody titers	No
Cooper, 2011 [39]	Canada	298 HIV-infected adults (90%:10%)	NR	Not measured	Not measured	Yes	TIV	A/Brisbane (H1N1); A/Uruguay (H3N2); B/Florida	Seroprotection Seroconversion	No
Chadha, 2011 [40]	USA	35 Prostate cancer patients (100%:0%)	68	Median	9 (26%)	Partial	TIV	A/New Caledonia/20/99 (H1N1); A/Wisconsin/67/2005 (H3N2); and B/Malaysia/2506/2004	Antibody titers Seroprotection	Positive
Kriesel, 1999 [41]	USA	175 healthy volunteers (52.6%:47.4%)	32	Not measured	Not measured	Yes	TIV	A/Wuhan/359/95(H3N2); A/Singapore/6/1986(H1N1); B/Beijing/184/93	Antibody titers Seroprotection	No

Abbreviations: AOM: acute otitis media; HIV: human immunodeficiency virus; LAIV: live attenuated influenza vaccine; NR: not reported; TIV: trivalent inactivated influenza vaccine; VDD: vitamin D deficiency; y/o = year-old. *: included in meta-analysis.

**Table 2:** Immunologic responses for influenza vaccination between VDD and control groups.

Study Population	Seroprotection Rate			Seroconversion Rate		
	A/H1N1	A/H3N2	B strain	A/H1N1	A/H3N2	B Strain
Vitamin D def	46.40%	71.80%	69.60%	34.20%	55.30%	29.90%
Control	47.70%	80.10%	76.40%	34.10%	56.40%	30.40%
Odds ratio	1 (0.52–1.92)	0.63 (0.43–0.91)	0.68 (0.5–0.93)	1.1 (0.79–1.54)	0.98 (0.76–1.25)	0.98 (0.75–1.27)
*p*	0.99	0.01	0.01	0.58	0.19	0.88

## Supplementary Materials

The following are available online at http://www.mdpi.com/2072-6643/10/4/409/s1, Table S1: Detailed search strategy of systematic review, Table S2: Risk of bias assessment of each included study.
