# Supplementary material for: Does Vitamin D Deficiency Affect the Immunogenic Responses to Influenza Vaccination? A Systematic Review and Meta-Analysis
Source: Nutrients. 2018 Mar 26;10(4):409. doi: 10.3390/nu10040409 (PMC5946194; doi:10.3390/nu10040409)
Supplement: Supplementary file 1 [file nutrients-10-00409-s001.zip › nutrients-283570-sp/Table-S1-Detailed-searching-strategy.docx]

Table S1. Detailed searching strategy of systematic review

PubMed

| ((((Flu Vaccine* OR Afluria OR Influenza Vaccine* OR Afluria OR Influenzavirus Vaccine* OR LAIV vaccine OR FluMist OR CAIV-T vaccine OR Trivalent Live Attenuated Influenza Vaccine OR Influenza Virus Vaccine*)) OR ((((Influenza, Human) OR (Influenza* OR flu)))  in All Fields |
| --- |
| AND |
| ((vaccination) OR vaccine*))))  in All Fields |
| AND |
| (((calcitriol) OR (Calcidiol) OR (1,25-dihydroxy-vitamin D3) OR ((25-(OH) D)) OR (25-hydroxyvitamin D) OR (vitamin D) OR (vit D) OR (vitamin d*) OR vitamin*)))  in All Fields |

Embase

| Influenza Vaccines OR Flu Vaccine* OR Afluria OR Influenza Vaccines OR Flu Vaccine* OR Afluria OR Influenzavirus Vaccine* OR LAIV vaccine OR FluMist OR CAIV-T vaccine OR Trivalent Live Attenuated Influenza Vaccine OR Influenza Virus Vaccine* |
| --- |
| AND |
| vaccination OR vaccine* |
| AND |
| (((calcitriol) OR (Calcidiol) OR (1,25-dihydroxy-vitamin D3) OR ((25-(OH) D)) OR (25-hydroxyvitamin D) OR (vitamin D) OR (vit D) OR (vitamin d*) OR vitamin*))) |

Cochrane Database

| Influenza Vaccines OR Flu Vaccine* OR Afluria OR Influenza Vaccines OR Flu Vaccine* OR Afluria OR Influenzavirus Vaccine* OR LAIV vaccine OR FluMist OR CAIV-T vaccine OR Trivalent Live Attenuated Influenza Vaccine OR Influenza Virus Vaccine* |
| --- |
| AND |
| vaccination OR vaccine* |
| AND |
| calcitriol OR Calcidiol OR 1,25-dihydroxy-vitamin D3 OR 25-(OH) D OR 25-hydroxyvitamin D OR vitamin D OR vit D OR vitamin d* OR vitamin* |

CINAHL Database (Cumulative Index to Nursing and Allied Health Literature Database)

| ((((Flu Vaccine* OR Afluria OR Influenza Vaccine* OR Afluria OR Influenzavirus Vaccine* OR LAIV vaccine OR FluMist OR CAIV-T vaccine OR Trivalent Live Attenuated Influenza Vaccine OR Influenza Virus Vaccine*)) OR ((((Influenza, Human) OR (Influenza* OR flu))) |
| --- |
| AND |
| ((vaccination) OR vaccine*)))) |
| AND |
| (((calcitriol) OR (Calcidiol) OR (1,25-dihydroxy-vitamin D3) OR ((25-(OH) D)) OR (25-hydroxyvitamin D) OR (vitamin D) OR (vit D) OR (vitamin d*) OR vitamin*))) |

Airiti (華藝數位，Art Image Indexing Service on the Internet Database)

| 流感 OR 流行性感冒  OR 感冒 |
| --- |
| AND |
| 疫苗 |
| AND |
| 維他命 OR 維他命D OR 維生素 OR 維生素D OR 骨化二醇 OR 骨化三醇 OR 1,25-二羥膽鈣化醇 OR [膽鈣化醇](https://zh.wikipedia.org/wiki/%E8%83%86%E9%92%99%E5%8C%96%E9%86%87) |

NTLTD (National Digital Library of Theses and Dissertations in Taiwan)

| 流感 + 流行性感冒 + 感冒 |
| --- |
| AND |
| 疫苗 |
| AND |
| 維他命 +維他命D +維生素+維生素D+ 骨化二醇+ 骨化三醇+ 1,25-二羥膽鈣化醇+ [膽鈣化醇](https://zh.wikipedia.org/wiki/%E8%83%86%E9%92%99%E5%8C%96%E9%86%87) |
