# Supplementary material for: Does Vitamin D Deficiency Affect the Immunogenic Responses to Influenza Vaccination? A Systematic Review and Meta-Analysis
Source: Nutrients. 2018 Mar 26;10(4):409. doi: 10.3390/nu10040409 (PMC5946194; doi:10.3390/nu10040409)
Supplement: Supplementary file 1 [file nutrients-10-00409-s001.zip › nutrients-283570-sp/Table-S2-Quality-assessment-of-included-studies-based-on-Cochrane-risk-of-Bias-tool.docx]

Table S2. Risk of bias assessment of each included study

| Study Validity  Domains | Sequence generation | Allocation Concealment | Blinding of participants and personnel and outcome assessors | Incomplete outcome data | Selective outcome reporting | Other sources  of bias |
| --- | --- | --- | --- | --- | --- | --- |
| Lin, 2017 | Low | Low | Low | High | Low | Uncertain |
| Sadarangani, 2016 | Low | Low | Low | High | Low | Uncertain |
| Crum-Cianflone, 2016 | Low | Low | Low | High | Low | Uncertain |
| Science, 2014 | Low | Unclear | Unclear | High | Low | Uncertain |
| Sundaram, 2013 | Low | Low | Low | High | Low | Uncertain |
| Principi, 2013 | Low | Low | Low | Low | Low | Low |
| Cooper, 2011 | Unclear | Unclear | High | Low | Uncertain | Uncertain |
| Chadha, 2011 | Low | Unclear | Unclear | Low | Uncertain | Uncertain |
| Kriesel, 1999 | Low | Low | Low | Low | Uncertain | Uncertain |

*. Each domain has been evaluated as being “High”, “Low”, or “Unclear” regarding the risk of bias following the guidelines of Cochrane Collaboration’s tool for assessing risk of bias “Low” in all Domains would place a study at “Low Risk of Bias”; “High” in any of the Domains would place a study at “High Risk of Bias”; “Unclear” in any of the domains would place the study at “Unclear Risk of Bias”
